# Coexistence of a novel NDM-1-encoding MDR plasmid and an IMP-4-encoding IncN-IncU hybrid plasmid in a clinical isolate of *Citrobacter freundii* BC73

**DOI:** 10.3389/fmicb.2024.1388651

**Published:** 2024-07-10

**Authors:** Na Liu, Biao Tang, Hui Wang, Xiangyang Chen, Peipei Wen, Zhaorui Wang, Xu Chen, Xiaobing Guo, Jianjun Gou, Yinsen Song

**Affiliations:** ^1^Translational Medicine Research Center, Zhengzhou People’s Hospital, The Fifth Clinical College of Henan University of Chinese Medicine, Zhengzhou, China; ^2^Key Laboratory of Systems Health Science of Zhejiang Province, School of Life Science, Hangzhou Institute for Advanced Study, University of Chinese Academy of Sciences, Hangzhou, China; ^3^Department of Laboratory Medicine, Zhengzhou People’s Hospital, The Fifth Clinical College of Henan University of Chinese Medicine, Zhengzhou, China; ^4^Department of Laboratory Medicine, The First Affiliated Hospital of Zhengzhou University, Zhengzhou, China

**Keywords:** *bla*_NDM-1_, *bla*_IMP-4_, *Citrobacter freundii*, MDR, genomics

## Abstract

**Objectives:**

To investigate the genetic characteristics and transmission mechanism of the NDM-1-, IMP-4-, and SHV-12-producing multidrug-resistant (MDR) clinical isolate, *Citrobacter freundii* BC73.

**Methods:**

*C. freundii* BC73 was isolated from a urine specimen of a urological patient diagnosed with bladder cancer at a Chinese teaching hospital. Antimicrobial susceptibility testing was carried out using DL-120E susceptibility cards and DL-96A system. Whole genome sequencing (WGS) of the isolate was performed using the Illumina and Oxford Nanopore platforms to analyze the genetic context of drug resistance genes and plasmid characteristics. The phylogenetic tree was constructed and visualized by KSNP3.0 software and iTOL5.0 online database.

**Results:**

*C. freundii* isolate BC73 co-carrying *bla*_NDM-1_, *bla*_IMP-4_ and *bla*_SHV-12_ were multidrug-resistant. *bla*_NDM-1_ and *bla*_IMP-4_ were located on a novel IncFIB-like plasmid, pCFBC1, and an IncN-IncU hybrid plasmid, pCFBC2, respectively. The transferability of *bla*_NDM-1_ and *bla*_IMP-4_ from *C. freundii* BC73 to *E. coli* J53 was successfully demonstrated. The genetic context of the *bla*_NDM-1_ and *bla*_IMP-4_ genes were IS*CR27*-*groEL*-*∆groES*-*cutA*-*dsbD*-*trpF*-*ble*_MBL_-*bla*_NDM-1_-∆IS*Aba125*-IS3000 and *intI1*-*bla*_IMP-4_-*Kl.pn.13*-*mobC*-IS*6100*, respectively. Additionally, two extensive transposition units (MGE1 in pCFBC1, MGE2 in pCFBC2) were identified and numerous antimicrobial resistance genes were discovered on it.

**Conclusion:**

To our knowledge, our study represents the first characterization of a ST22 *C. freundii* isolate co-harboring *bla*_NDM-1_, *bla*_IMP-4_, and *bla*_SHV-12_, obtained from a urine sample. The dissemination of this MDR isolate should be of close concern in future clinical surveillance.

## Introduction

Infections due to carbapenemase-producing *Enterobacteriaceae* (CPE) remain pose a major threat to the public health (Nordmann et al., 2011; Tang et al., 2023). In particular, the co-production of two or three carbapenemases in a single bacterial isolate has become increasingly prevalent over the past 5 years, and resistance has shown an increase compared to the presence of a single gene. Such as in the study by Biez et al., the MICs of imipenem, meropenem and ertapenem in *bla*_NDM-1_-*E. coli* J53 or *bla*_OXA-48_-*E. coli* J53 transconjugants (Tc) or *bla*_VIM-1_-*E. coli* TOP10 transformant (Tf) were significantly lower than the original strain NDM-1-, VIM-1- and OXA-48-producing *C. freundii* 255A1. In another report, the MIC of meropenem in the original strain 112,298 was the same as the highest MIC in the transformants (112298-KPC-TOP10 and 112,298-NDM-TOP10) (Feng et al., 2015; Biez et al., 2022). We should beware of the emergence of such strains. The *bla*_NDM-1_ and *bla*_IMP-4_ genes, both encoding metallo-beta-lactamases (MBLs) with high carbapenemase activity, enable them to hydrolyze nearly all β-lactams including carbapenems. In recent years, they have been frequently detected in a diverse array of gram-negative bacteria, leading to the occurrence of numerous serious outbreaks (Yong et al., 2009; Dolejska et al., 2016; Xiong et al., 2016; Matsumura et al., 2017; Guducuoglu et al., 2018; Roberts et al., 2020). The simultaneous presence of these two resistance genes in a single strain may result in the emergence of highly drug-resistant variants, presenting a significant challenge for the treatment of infections.

*Citrobacter freundii*, a member of *Enterobacteriaceae* family and widely existed in water, soil, and the intestines of both animals and humans, has been identified as an opportunistic pathogen responsible for various infections including urinary, gastrointestinal, respiratory, peritoneal and bloodstream infections (Bodey, 2005). Unfortunately, the indiscriminate use of carbapenems has led to an escalating acquired resistance to antibiotics in *C. freundii* in recent years. So far, carbapenemases such as KPC-2-, NDM-1-, IMP-4-, OXA-48- and VIM-1-type have been reported in *C. freundii*, with affected regions including China, India, Spain, France and Italy (Yong et al., 2009; Gaibani et al., 2013; Feng et al., 2015; Lalaoui et al., 2019; Biez et al., 2022). However, the coexistence of NDM-1 and IMP-4 in single *C. freundii* isolate, along with its characteristics of transmission and resistance, has been rarely documented.

In this study, we identified a ST22 isolate of *C. freundii*, named BC73, which is the first reported case of co-carrying *bla*_NDM-1_, *bla*_IMP-4_ and *bla*_SHV-12_ from urine. Upon comprehensive investigation, we discovered that *bla*_NDM-1_ and *bla*_IMP-4_ were carried by a novel MDR plasmid and an IncN-IncU hybrid plasmid, respectively. Additionally, two extensive transposition units (MGE1 in pCFBC1, MGE2 in pCFBC2) harboring multiple resistance genes were identified, which were a potential contribution to the dissemination of multiple drug resistance.

## Materials and methods

### Bacterial isolation and susceptibility testing

A urine specimen was obtained from a hospital patient undergoing examination at the Fifth Clinical Medical College of Henan University of Chinese Medicine (FCMC-HUCM), Zhengzhou, China, in December 2021. The sample were cultured on MacConkey agar (OXOID, Hampshire, United Kingdom) plates supplemented with 2 mg/L meropenem and incubated at 37°C for 18–24 h. Species identification was conducted using matrix-assisted laser desorption/ionization time-of-flight mass spectrometry (MALDI-TOF/MS) (Bruker, Bremen, Germany) and 16S rRNA gene sequencing. *In vitro* susceptibility test was performed using DL-120E susceptibility cards and DL-96A system (Zhuhai Deere Biological Engineering Co., LTD), which included 25 antibacterial agents as listed in Table 1. The interpretation of results followed the guidelines of the Clinical Laboratory Standards Institute (CLSI 2021; Humphries et al., 2021), with the exceptions of tigecycline and colistin, for which clinical breakpoints were determined according to the U.S. Food and Drug Administration (FDA) (Marchaim et al., 2014) and the European Committee on Antimicrobial Susceptibility Testing (EUCAST, 2022) guidelines^1^, respectively. Ultimately, *C. freundii* BC73 was confirmed and its details were presented in the subsequent results.

**Table 1:** The results of antimicrobial susceptibility testing.

Antibiotic class/Antibiotics	*C. freundii* BC73		Transconjugant		*E. coli* J53	
	MIC (mg/L)	R/I/S	MIC (mg/L)	R/I/S	MIC (mg/L)	R/I/S
β-lactams						
Ampicillin	>16	R	>16	R	<8	S
Ampicillin/Sulbactam	>16/8	R	>16/8	R	<8/4	S
Piperacillin/tazobactam^a^	>64/4	R	>64/4	R	<4/4	S
Cefoperazone/Sulbactam	>64/32	R	>64/32	R	<8/4	S
Ceftazidime/Clavulanic Acid	>1/4	R	>1/4	R	<1/4	S
Cefotaxime/Clavulanic Acid	>1/4	R	>1/4	R	<1/4	S
Cefazolin	>16	R	>16	R	<2	S
Cefepime	16	R	>16	R	<0.12	S
Cefoxitin	>32	R	>32	R	<8	S
Cafuroxime	>32	R	>32	R	8	S
Ceftazidime	>16	R	>16	R	<0.5	S
Cefotaxime	>32	R	>32	R	<0.12	S
Imipenem	4	R	16	R	<0.25	S
Meropenem	16	R	16	R	<0.06	S
Ertapenem	>8	R	>8	R	<0.015	S
Fluoroquinolone						
Levofloxacin	>4	R	<0.12	S	<0.12	S
Sulfonamide						
Trimethoprim/sulfamethoxazole	>4/76	R	<0.5/9.5	S	<0.5/9.5	S
Phenicol						
Chloramphenicol	>16	R	>16	R	<8	S
Nitrofurans						
Nitrofurantoin	<16	S	<16	S	<16	S
Tetracycline						
Minocycline	>8	R	>8	R	<1	S
Tigecycline	<0.25	S	<0.25	S	<0.25	S
polymyxin						
Polymyxin B	<1	S	<1	S	<1	S
macrolide						
Azithromycin	>32	R	>32	R	<8	S
Aminoglycosides						
Gentamicin	>8	R	>8	R	<1	S
Amikacin	<4	S	<4	S	<4	S

R, resistant; I, Intermediary; S, susceptible.

a Tazobactam at a fixed concentration of 4 mg/L.

### Transferability of plasmids carrying *bla*_NDM-1_ and *bla*_IMP-4_, respectively

The *bla*_NDM/IMP_-carrying plasmids were visualized through PFGE/S1 nuclease analysis, followed by southern hybridization, utilizing digoxigenin-labeled *bla*_NDM-1_ and *bla*_IMP-4_-specific probes. The conjugation transfer of plasmids was executed by co-culturing with the recipient *E. coli* J53 at a 1:10 donor-to-recipient ratio, maintained at 25°C (Gou et al., 2020). Transconjugants were selectively cultivated on Mueller-Hinton medium supplemented with sodium azide (150 mg/L) and meropenem (2 mg/L). The confirmation of the selected transconjugants were carried out through PCR experiments.

### Whole-genome sequencing and data analysis

Total DNA was extracted utilizing the Tiangen Genomic DNA Extraction Kit (Tiangen, Beijing, China) and subsequently sequenced using the Illumina HiSeq 4,000-PE150 (Illumina, San Diego, United States) and Oxford Nanopore GridION (Nanopore, Oxford, United Kingdom) platforms. *De novo* assembly of both short reads and long reads was conducted using Unicycler v0.4.8 (Wick et al., 2017), and the genomic sequences were annotated through the NCBI prokaryotic genome annotation pipeline. To identify sequence types (ST) and antimicrobial resistance genes, PubMLST^2^ and ResFinder 4.5^3^ were employed. Replicon types of plasmid were performed by PlasmidFinder 2.1.^4^ The conjugation transfer modules in plasmids were predicted by oriTfinder^5^ and ICEfinder.^6^ Virulence factors (VFs) and mobile genetic elements (MGEs) were identified using VRprofile 2.^7^ CRISPR arrays were conducted by CRISPR Finder.^8^ Additionally, sequence comparisons were executed using BLAST.^9^ Comparative maps of the gene environment surrounding *bla*_NDM-1_ and *bla*_IMP-4_ genes were generated by Easyfig (Sullivan et al., 2011) and the BLAST Ring Image Generator (BRIG) (Alikhan et al., 2011) tool.

### Phylogenetic analysis

Genome sequences of 45 available *C. freundii* isolates were downloaded from the NCBI^10^ database, with *C. freundii* B38 (GCA_001702455.1) selected as the reference genome for comparative analysis. Subsequently, *C. freundii* BC73 and other *C. freundii* genomes were analyzed based on core genomic single nucleotide polymorphisms (SNPs) using KSNP3.0 (Gardner et al., 2015). Finally, a maximum likelihood tree was generated and visualized by iTOL5.0 (Letunic and Bork, 2021).

### GenBank accession numbers

The complete genome sequence of *C. freundii* BC73 has been submitted to GenBank and assigned the accession numbers CP117475-CP117478.

## Results

### Clinical *C. freundii* BC73 co-carrying *bla*_NDM-1_ and *bla*_IMP-4_

Carbapenem-resistant *C. freundii* BC73 was isolated from a urine specimen of the patient who was hospitalized for urinary tract infection. The patient had a history of bladder cancer and had undergone total cystotomy and abdominal fistula drainage three months prior. Subsequent PCR and sequencing confirmed that the isolate was *C. freundii* carrying both *bla*_NDM-1_ and *bla*_IMP-4_ (Supplement 1).

### Antimicrobial susceptibility testing

**Figure 1:**
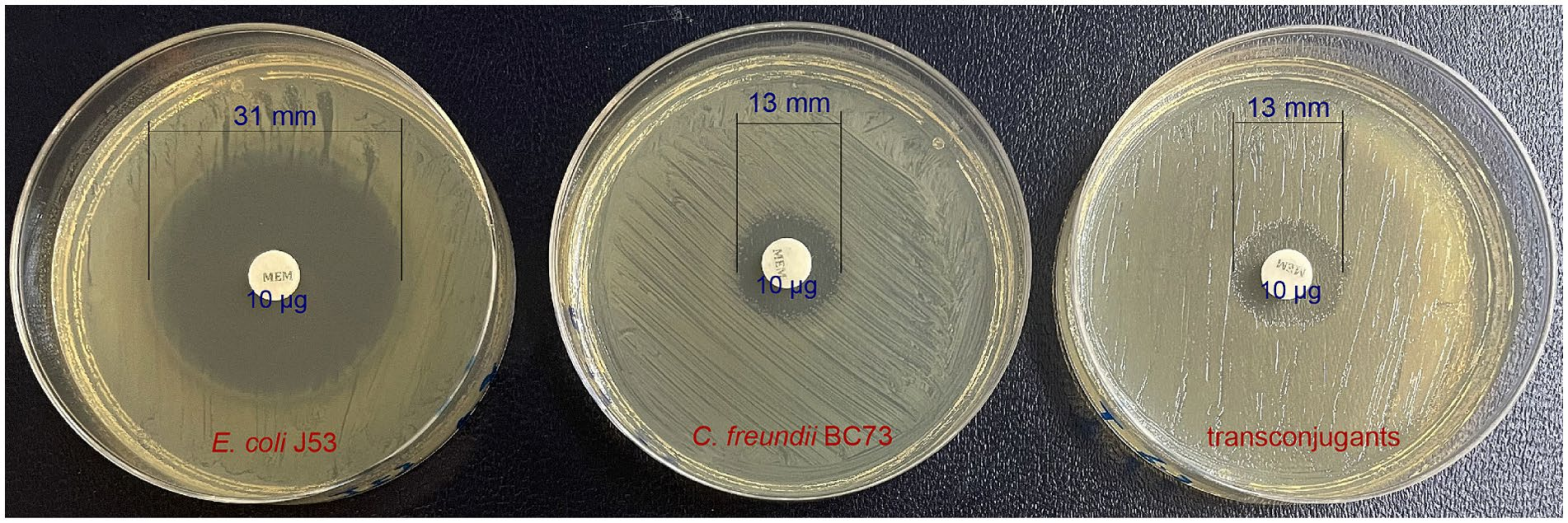
The results of the inhibition zones summarizing the measurements.

The antimicrobial susceptibility results were presented in Table 1 and the image of the inhibition zones was deposited in Figure 1. Both *C. freundii* BC73 and transconjugant BC73-J53 exhibited resistance to all β-lactams, chloramphenicol, minocycline, azithromycin, and gentamicin antibiotics tested while they were sensitive to nitrofurantoin, tigecycline, polymyxin B, and amikacin. In addition, when *C. freundii* BC73 was resistant to levofloxacin and trimethoprim/sulfamethoxazole, transconjugant BC73-J53 was sensitive to it. Interestingly, the resistance of transconjugant BC73-J53 to Imipenem and cefepime was greater than that of *C. freundii* BC73 to it. The recipient *E. coli* J53 was susceptible to all antibacterial agents tested.

### Genomic features of *Citrobacter freundii* BC73

The isolate BC73 was identified as ST22. The genome was a single chromosome spanning 5,160,079 bp, exhibiting an average G + C content of 51.6%. Additionally, three plasmids (pCFBC1, pCFBC2, pCFBC3) were identified. The chromosome possessed 4,973 coding genes, 29 ISs, and the virulence genes *csgABC*, *rcsA*, *wbtL*, *misL*, and *galE*. Notably, anti-microbial resistance (AMR) genes *bla*_CMY-48_, *aadA1* and *dfrA1* were located on chromosome while *bla*_NDM-1_ on pCFBC1 and *bla*_IMP-4_ on pCFBC2 (Table 2).

**Table 2:** Overall features of the *C. freundii* BC73 Genome.

Parameter	*C. freundii* BC73			
	Chromosome	pCFBC1	pCFBC2	pCFBC3
Size(bp)	5,160,079	130,842	68,426	4,942
G + C (%)	51.6	53.1	51.4	49.9
MLST	ST22	–	–	–
Number of coding genes	4,973	156	99	9
Number of ISs	29	26	6	–
Virulence genes	csgABC, rcsA, wbtL, misL, and galE	–	–	–
CRISPR arrays	1	–	–	–
Resistance determinants	bla_CMY-48_, aadA1, dfrA1	bla_NDM-1_, bla_SHV-12_, bla_DHA-1_, qnrB4, aac(3)-IId, sul1, sul2, tet(D), qacE∆1, mph(A), catA2, catB3, arr-3, chrA	bla_IMP-4_, aac(6′)-Ib3, qacE∆1, qnrS1, arr-3	–
Plasmid types (Inc)	–	IncFIB-like	IncU-IncN	–
Accession numbers	CP117475	CP117476	CP117477	CP117478

### Characterization of pCFBC1 and pCFBC2

**Figure 2:**
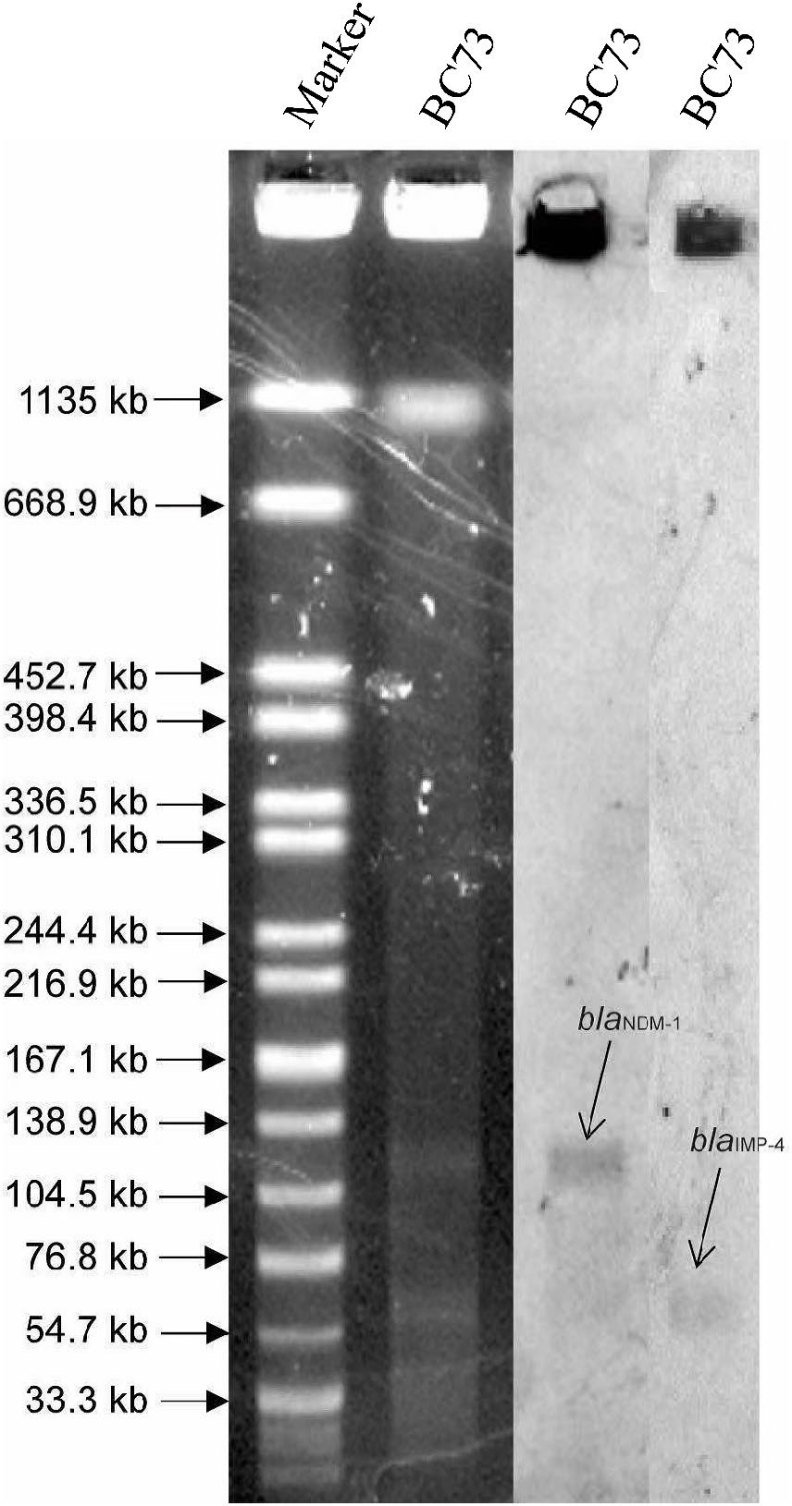
The identification of plasmids size using S1-PFGE (left) and southern blot and hybridization (right). pCFBC1 plasmid was between 104.5 kb and 138.9 kb, which was positive for a probe against *bla*_NDM-1_. pCFBC2 plasmid was between 54.7 kb and 76.8 kb, which was positive for a probe against *bla*_IMP-4_.

**Figure 3:**
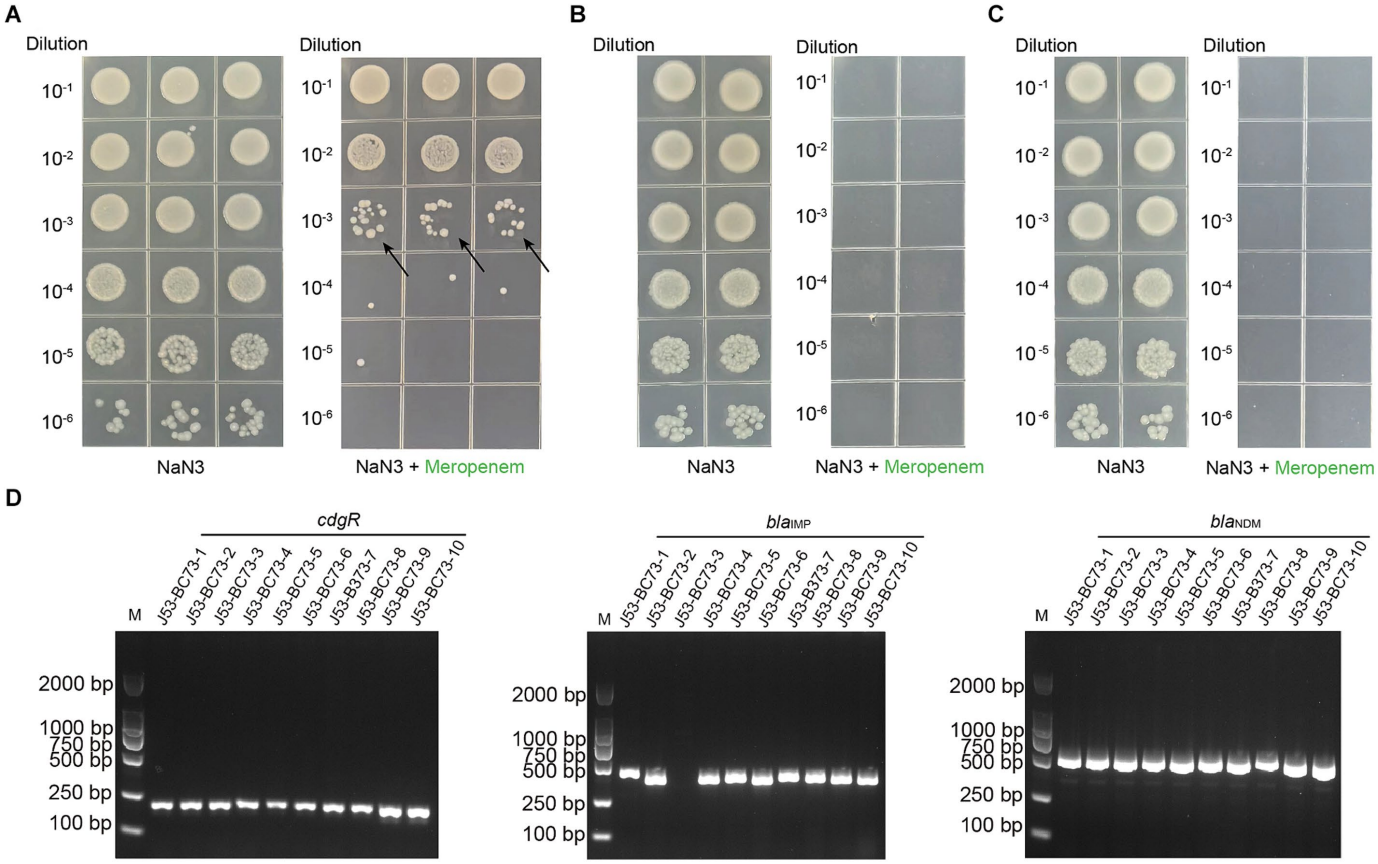
Conjugation transfer test and PCR verification of transconjugants of *C. freundii* strain BC73 with sodium azide-resistant *E. coli* strain J53 served as the recipient strain. **(A)** Conjugation transfer test of *C. freundii* strain BC73. From left to right are the screening results under the selective pressure of sodium azide and meropenem. The arrow represents the transconjugants. (B&C) Testing the transformation of the strain BC73’s genome DNA into the natural state of *E. coli* J53. One microliter of DNA at a concentration of 100 **(B)** & 1 **(C)** micrograms per milliliter was mixed with the BC73 strain and two repetitions were made. **(D)** PCR verification of transconjugants with sodium azide and meropenem. *cdgR* gene is a specific primer for *E. coli*.

**Figure 4:**
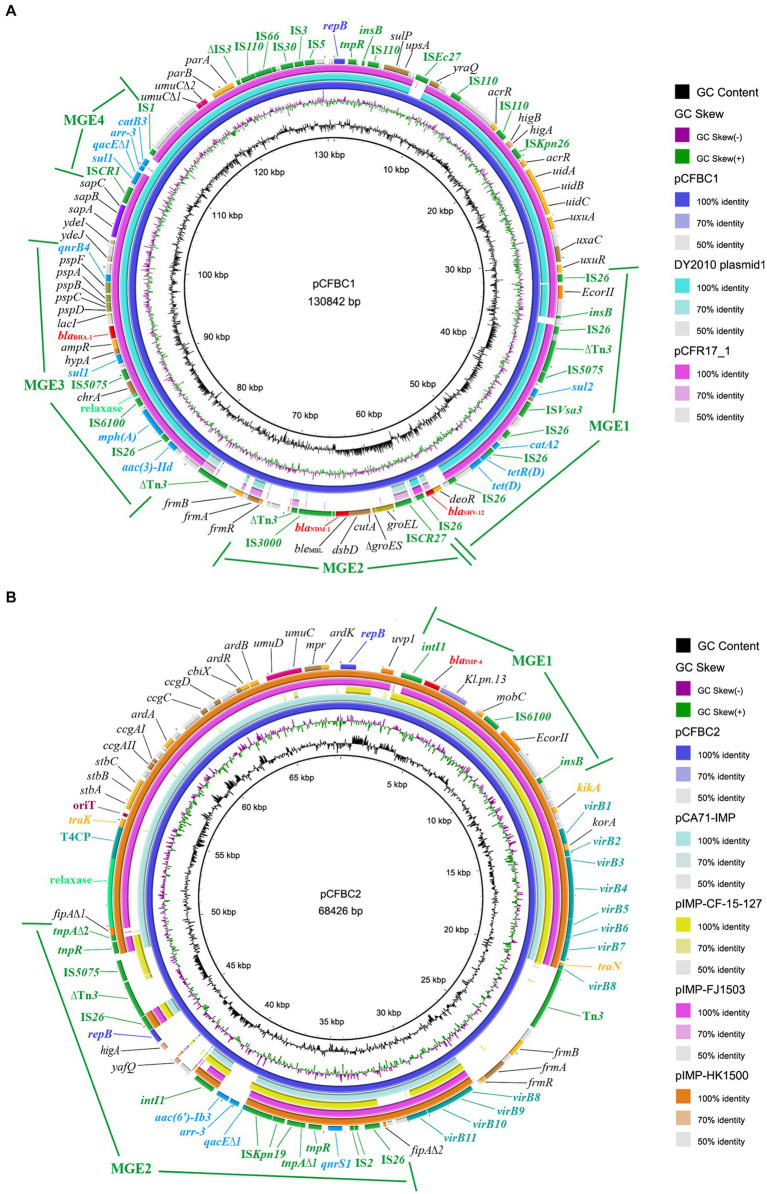
Plasmid comparison of pCFBC1 and pCFBC2 plasmids. **(A)** Comparison of the pCFBC1 with DY2010 plasmid 1 (CP086288) and pCFR17_1 (CP035277). **(B)** Comparison of the pCFBC2 with pCA71-IMP (CP064181), pIMP-CF-127 (CP068026), pIMP-FJ1503 (KU051710) and pIMP-HK1500 (KT989599).

*C. freundii* BC73 carried a ~131 kb plasmid harboring *bla*_NDM-1_ gene and a ~68 kb plasmid encoding *bla*_IMP-4_ gene (Figure 2). pCFBC2 was successfully transferred to *E. coli* J53 from *C. freundii* BC73 by conjugative assays and the conjugative efficiency was (1.11 ± 0.29) × 10^−3^ (Figure 3). pCFBC1 was a novel nonseparable plasmid, designated as IncFIB-like plasmid, with 130,842 bp in length and an average GC content of 53.1% (Table 2). A collection of replication initiation and stability proteins (*repB*, *parAB*), transcriptional regulators (*acrR*, *deoR*, *frmBR*, *uidABC*, *uxuAR*, *ampR*, *lacI*) formed the backbone of pCFBC1. Furthermore, four mobile genetic elements (MGEs) including MGE1, MGE2, MGE3 and MGE4 were found in this plasmid. In these MGEs, a lot of transposition units comprising ISs and antimicrobial resistance genes such as IS*26-aac(3)-IId* module, IS*6100*-*mph(A)-mrx-mphR* module, *chrA*-IS*5075*-*sul1* module, IS*CR1*-*sul1*-*qacE∆1*-*arr-3*-*catB3*-IS1 module, and IS26-based module (IS*26*-*bla*_SHV-12_-IS*26*-*tet(D)*-IS*26*-*catA2*-IS*26*-IS*Vsa3*-*sul2*-IS*5075*-∆Tn*3*-IS*26*-*insB*-IS*26*) were found. In comparison with selected plasmids, pCFBC1 exhibited 100.00 and 99.99% nucleotide identity with DY2010 plasmid 1 (CP086288) and pCFR17_1 (CP035277), respectively (Figure 4A). On the other hand, pCFBC2 emerged as an IncN and IncU hybrid plasmid, with 68,426 bp in length and an average GC content of 51.4% (Table 2). It featured two *repB*, *mobC*, *frmBR*, *stbABC* and *ardABKR* genes essential for replication and maintenance. In addition, the complete system for conjugation transfer including *traKN*, *kikA*, *oriT*, relaxase, the type IV coupling proteins (T4CP) and the type IV secretion system (T4SS) (*virB1*-11) was found. Two variable regions (VR1 and VR2) including a *bla*_IMP-4_ associated In*823* and an extensive transposition unit (MGE2 in pCFBC2) were identified. These two regions harbored antimicrobial resistance genes including *bla*_IMP-4_, *aac(6′)-Ib3*, *qacE∆1*, *qnrS1*, and *arr-3*. pCFBC2 exhibited 99.97% nucleotide identity with pCA71-IMP (CP064181) and pIMP-HK1500 (KT989599), as detailed in Figure 4B.

### Genetic environment of *bla*_NDM-1_ and *bla*_IMP-4_ genes

**Figure 5:**
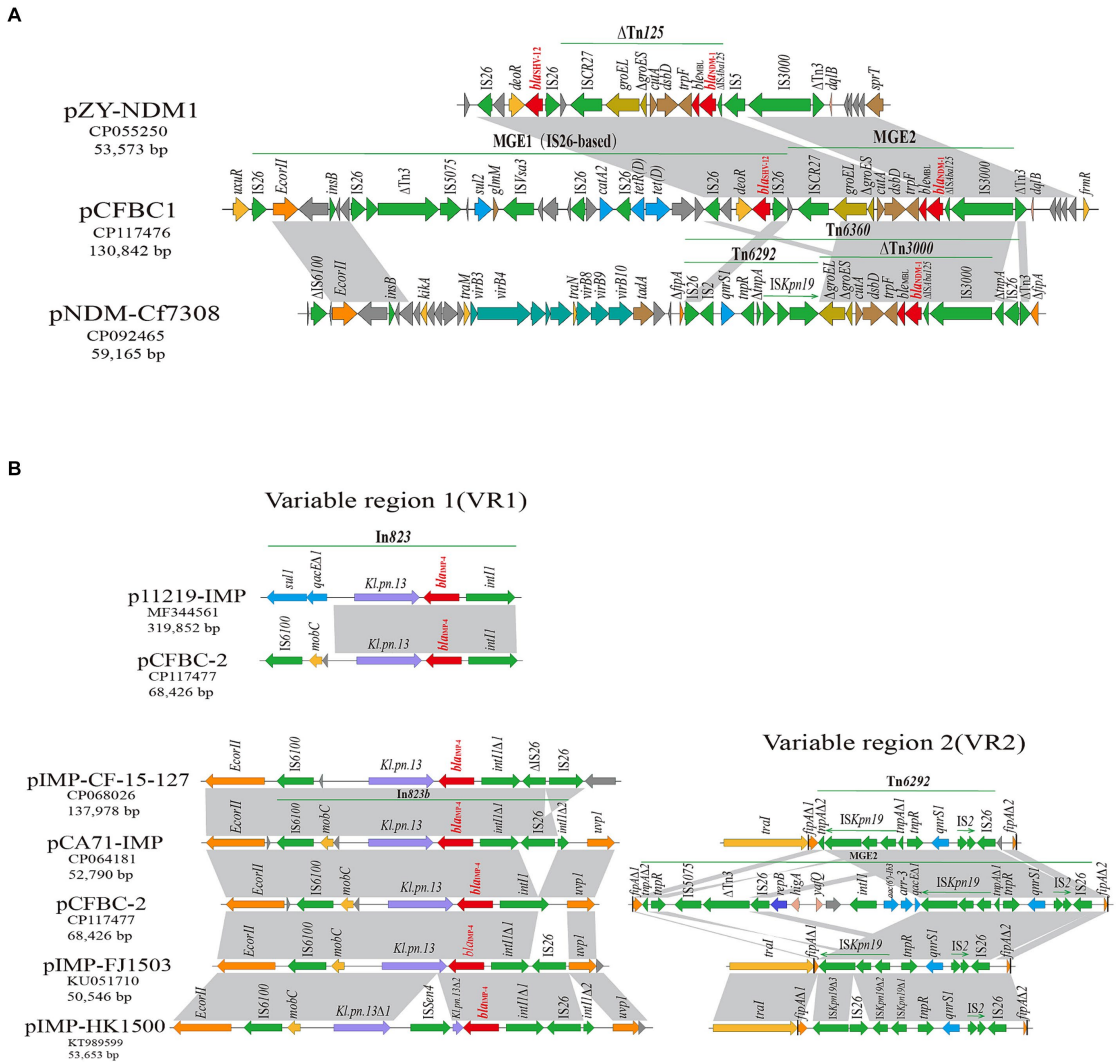
Gene-environment comparison of *bla*_NDM-1_ and *bla*_IMP-4_. **(A)** Genetic environment of *bla*_NDM-1_ on pCFBC1 and related plasmids. The regions with highly similar sequences were marked by light gray. The *bla*_NDM-1_ genes were shown by red; mobile elements were drawn by green. **(B)** In VR1, genetic context of *bla*_IMP-4_ on pCFBC2 and related plasmids. The *bla*_IMP-4_ genes were shown by red. In VR2, the genetic feature of Tn*6292*-associated area on pCFBC2 and related plasmids.

In pCFBC1, the *bla*_NDM-1_ gene resided within the composite structure of ∆Tn*3000* and ∆Tn*125* (IS*CR27*-*groEL* -*∆groES*-*cutA*-*dsbD*-*trpF*-*ble*_MBL_-*bla*_NDM-1_-∆IS*Aba125*-IS*3000*). Upstream of this structure was MGE1 (IS26-based), comprised of a transposon from the Tn*3* family, insertion sequences IS26, IS*5075* and IS*Vsa3* and antimicrobial resistance genes (*bla*_SHV-12_, *tet(D)*, *catA2*, *sul2*). Sequence comparisons revealed minor variations in the immediate genetic context of *bla*_NDM-1_ among the three plasmids (pCFBC1, pZY-NDM1, pNDM-Cf7308) (Figure 5A). In VR1 of pCFBC-2 (Figure 5B), the class 1 integron In*823* carrying the resistance gene cassette *bla*_IMP-4_, the group II intron *Kl.pn.13* and a mobilization protein (*mobC*) was inserted between the *EcorII* and *uvp1* gene. And IS*6100* was inserted downstream of the integron. In VR2 of pCFBC-2 (Figure 5B), the *fipA gene* was interrupted by the insertion of a ~17 kb region (MGE2) including Tn*6292*, splitting it into two fragments (*fipA∆1* and *fipA∆2*). Tn*6292* consisted of relics of Tn*6292 tnp* genes (*tnpA* and *tnpR*), insertion sequences (IS*Kpn19*, IS*2*-IS*26*), and the *qnrS1* gene. Interestingly, *tnpA* was disrupted and divided into two parts (*tnpA∆1* and *tnpA∆2*) by IS*Kpn19* and a ~13 kb complex sequence, which was flanked by mobile elements *tnpR*-IS*5075*-∆Tn*3-*IS*26* and a class 1 integron structure: *intI1*-*aac(6′)-Ib3*-*arr-3*-*qacE∆1*, lacking a common *sul1* gene. In the middle, a replication initiation protein *repB* and two toxin-antitoxin proteins (*higA*, *yafQ*) were identified. A linear comparison of the *bla*_IMP-4_ genetic background among these plasmids displayed several differences: (1) the integrase gene *intI1* immediately upstream of *bla*_IMP-4_ was complete in p11219-IMP (MF344561) and pCFBC-2, but interrupted by IS*26* in pCA71-IMP, pIMP-*CF*-15-127 (CP068026), pIMP-FJ1503 (KU051710), and pIMP-HK1500. (2) The group II intron *Kl.pn.13* immediately downstream of *bla*_IMP-4_ was disturbed by IS*Sen4* only in pIMP-HK1500. (3) A common 3′-conserved segment *(qacE∆1*-*sul1*) of class 1 integron In*823* in p11219-IMP was absent in others plasmid and IS*6100* was inserted at the far-end downstream of *bla*_IMP-4_. (4) There was no IS*26* upstream of *bla*_IMP-4_ only in p11219-IMP and pCFBC-2 (VR1 of Figure 5B). Additionally, due to the fragmentation and rearrangement of genetic content, different variants of Tn*6292* were generated in four plasmids, but the most significant variation was found in pCFBC-2, reflecting in a complex sequence inserted above-mentioned (VR2 of Figure 5B).

### Phylogenetic analysis

**Figure 6:**
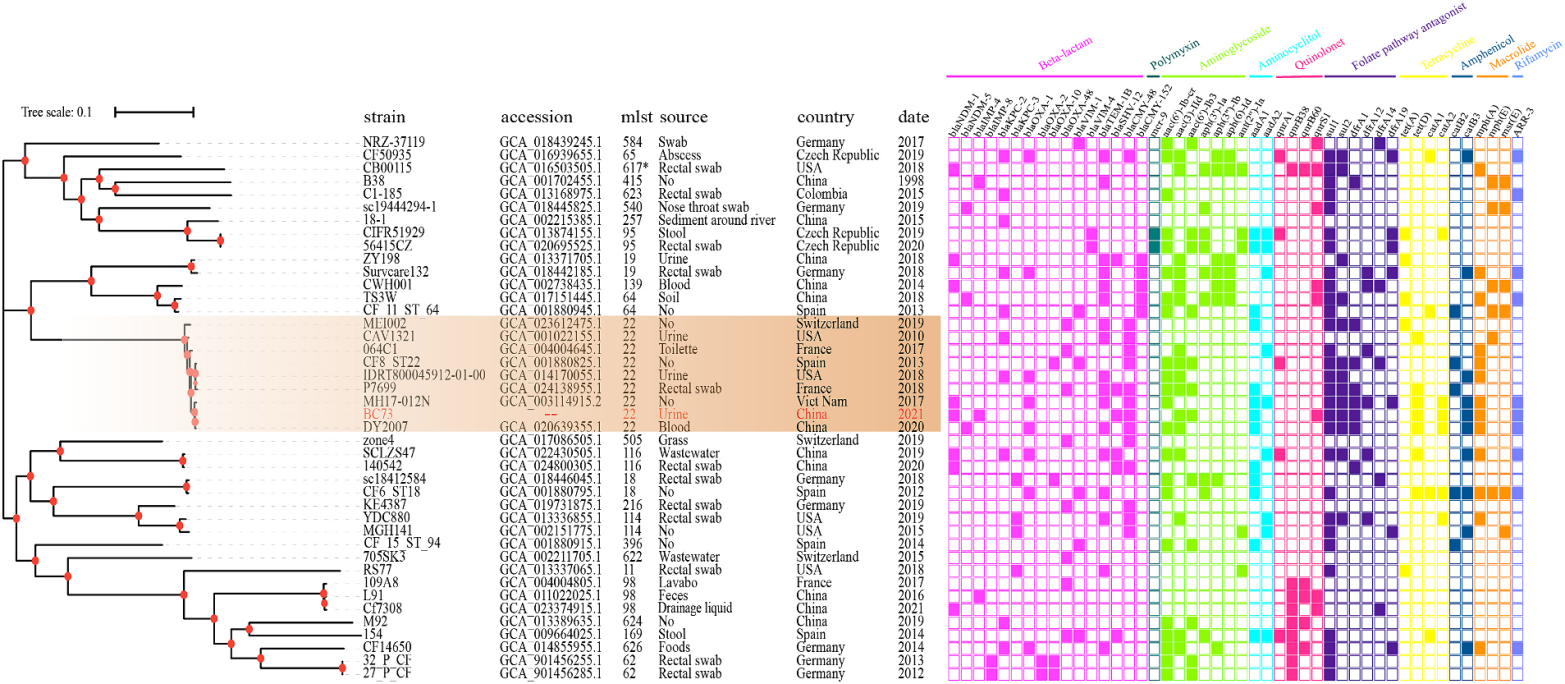
The phylogenetic tree composed of 46 *C. freundii* genome generated by KSNP3.0, where *C. freundii* B38 was used as the standard strain. Different color of the right panel indicated different positive antibiotic resistance genes of the corresponding strains.

The diverse STs of 46 *C. freundii* isolates were displayed in Figure 6, which mainly included 22, 98, 116, 64, 396. These isolates, obtained from various specimen types including rectal swab, urine, stool, blood, abscess, nose throat swab, drainage liquid, lavabo, toilette, wastewater, soil, grass, sediment around river and food, were sourced from different hosts (homo sapiens, environment, food) across multiple countries including China, Germany, France, Spain, Switzerland, United States, Czech Republic, and Viet Nam spanning the period from 1998 to the present. The majority of *C. freundii* isolates carried resistance determinants such as β-lactams (*bla*_NDM_, *bla*_KPC_, *bla*_VIM_, *bla*_IMP_, *bla*_OXA_, *bla*_SHV_, *bla*_TEM_, *bla*_CMY_), aminoglycosides (*aac(6′)-Ib-cr*, *aac(3)-IId*, *aac(6′)-Ib3*, *aadA1*, *aadA2*), and folate pathway antagonists (*sul1*, *sul2*). Furthermore, *C. freundii* BC73 exhibited clustering with *C. freundii* MEI002, *C. freundii* CAV1321, *C. freundii* 064C1, *C. freundii* CF8_ST22, *C. freundii* IDR1800045912-01-00, *C. freundii* P7699, *C. freundii* MH17-012 N, and *C. freundii* DY2007. Notably, *C. freundii* DY2007, encoded *bla*_NDM-5_ and *bla*_OXA-1_ genes, was isolated from a blood specimen in China in 2020 and demonstrated the closest relationship to *C. freundii* BC73.

## Discussion

*Citrobacter freundii* resistant to carbapenems has been gradually observed in patients with hospital-acquired infections (Hammerum et al., 2016; Zhang et al., 2023). However, studies on its transmission mechanisms and resistance characteristics, especially in case involving *C. freundii* carrying multiple carbapenemase genes, were scarce. In the routine collection of CRE strains, we obtained the *C. freundii* isolate BC73 co-carrying the carbapenemase genes *bla*_NDM-1_, *bla*_IMP-4_, and ESBLs gene *bla*_SHV-12_. It was isolated from the urine of a 64-year-old patient with urinary tract infections resulting from total cystotomy and abdominal fistula drainage. Carbapenems resistant *Citrobacter freundii* isolates, especially multidrug-resistant strains, were gradually being found in urinary tract infections, which may cause the extension of infection and the increase of patients’ suffering (Prussing et al., 2020; Zhang et al., 2021; Ye et al., 2023). The AST results presented in Table 1 showed that *C. freundii* BC73 and transconjugant BC73-J53 were resistance to all β-lactams, chloramphenicol, minocycline, azithromycin, and gentamicin antibiotics tested. Conversely, they were susceptible to nitrofurantoin, tigecycline, polymyxin B, and amikacin. The resistant phenotype of *C. freundii* BC73 was consistent with resistant genotype of it, implying resistance determinants were likely responsible for multiple drug resistance.

Recently, IncX3 plasmids carrying *bla*_NDM_ have commonly been identified in different species of the *Enterobacteriaceae* (Ye et al., 2023), implying their significant role in *bla*_NDM_ transfer. Furthermore, a study from Zhang et al. showed a high transfer frequency were observed in pZY-NDM1, a IncX3 plasmid harboring *bla*_NDM-1_ gene (Zhang et al., 2021). Interestingly, we identified a nonseparable plasmid, pCFBC-1, co-carrying *bla*_NDM-1_, *bla*_SHV-12_ and *bla*_DHA-1_. In this plasmid, we found three noteworthy features: (1) pCFBC-1 (~131 kb) was a novel large plasmid carrying *bla*_NDM-1_ gene. Firstly, compared to the most homologous plasmids in Figure 4A, we speculated that pCFBC1 underwent a genetic recombination and a conserved structure (*groEL*-*∆groES*-*cutA*-*dsbD*-*trpF*-*ble*_MBL_-*bla*_NDM-1_-∆IS*Aba125*-IS*3000*) was recombined into pCFBC1. To our knowledge, this was the first time that the *bla*_NDM-1_ gene was present in this type plasmid and the resistance of it may be increased. Second, the most homologous plasmids of pCFBC1 have not been systematically analyzed. Based on the above, we believed that pCFBC1 was a novel plasmid. (2) After comparison with the PlasmidFinder database, pCFBC1 was unable to obtain the replicon type of the plasmid and was defined as a nonseparable plasmid. According to the replicon type of the plasmid with higher homology to pCFBC1, we named it InFIB-like plasmid. (3) Lots of transposition units (ISs + resistant determinants) were discovered in MGEs of pCFBC-1 such as IS*26-aac(3)-IId* module (aminoglycosides resistance), IS*6100*-*mph(A)-mrx-mphR* module (macrolides resistance), *chrA*-IS*5075*-*sul1* module (chromates and folate pathway antagonists resistance), IS*CR1*-*sul1*-*qacE∆1*-*arr-3*-*catB3*-IS*1* module (folate pathway antagonists resistance, quaternary ammonium compounds, rifamycin and phenicol resistance) and IS*26*-based module (IS*26*-*bla*_SHV-12_-IS*26*-*tet(D)*-IS*26*-*catA2*-IS*26*-IS*Vsa3*-*sul2*-IS*5075*-∆Tn*3*-IS*26*-*insB*-IS26/β-lactams, tetracyclines, phenicol and folate pathway antagonists resistance) (Figure 4A). The presence of IS*26* was highlighted as a potential contributor to the dissemination of resistance genes (Jia et al., 2022; Li et al., 2022). Wang et al. ever emphasized that genes encoding resistance could be recruited into a variable genetic locus flanked by IS elements and transposons, facilitating their common transfer in *Enterobacteriaceae* (Wang et al., 2017a). Additionally, three reports demonstrated (Toleman et al., 2006; Liu et al., 2015; Li et al., 2020) that IS*CR1* may contribute to the mobilization of *bla*_NDM-1_ through rolling-circle transposition, manifesting the potential of IS*CR1* in transferring resistance genes. At present, while *bla*_IMP-4_ has been found in various plasmid types (N, HI2, L/M and A/C), the IncN type, known for its broad-host-range and self-conjugative properties, remains predominant for the spread of *bla*_IMP-4_ in China (Lai et al., 2017; Wang et al., 2017b; Liu et al., 2021). In our study, we identified a IncN-IncU hybrid plasmid, pCFBC-2, carrying *bla*_IMP-4_ and a class 1 integron In*823*. The elements related to conjugation transfer such as *traKN*, *kikA*, *oriT*, relaxase, the type IV coupling proteins (T4CP) and the type IV secretion system (T4SS) (*virB1*-*11*) were revealed in pCFBC2. Furthermore, an extensive transposition unit (MGE2 in pCFBC2) were described. Compared to the most homologous IncN plasmids in Figure 4B, pCFBC2 not only possessed both IncN and IncU replicon types, but also added Tn3 family, *aac(6′)-Ib3* and *arr-3* on it, making its host range wider and mobility more flexible.

Horizontal gene transfer (HGT) plays a crucial role in the dissemination of bacterial resistance. The primary vehicle of HGT included plasmids, transposons (Tn), insertion sequences (IS) and integrons (In), which possessed the capability to capture and recombine genes associated with antibiotic resistance, heavy metal resistance and virulence, disseminating them with mobile characteristics (Qiao et al., 2023). Our investigation revealed that *C. freundii* BC73 successfully transferred *bla*_NDM-1_ and *bla*_IMP-4_, along with a carbapenem non-susceptible phenotype, to the recipient *E. coli* J53. This confirmed the natural horizontal gene transfer characteristic across species for *bla*_NDM-1_ and *bla*_IMP-4_. From the gene point of view, pCFBC2 had the complete conjugation transfer system, which further verified its autonomous conjugation transfer ability. According to our experimental validation in Figure 3, although pCFBC1 lacked elements related to conjugation transfer except part of relaxase, *bla*_NDM-1_ gene in it can be transferred to the recipient *E. coli* J53, suggesting that pCFBC1 may have been transferred to the recipient *E. coli* J53 with the help of pCFBC2. In pCFBC-1, *bla*_NDM-1_ was located in a conserved structure: IS*CR27*-*groEL*-*∆groES*-*cutA*-*dsbD*-*trpF*-*ble*_MBL_-*bla*_NDM-1_-∆IS*Aba125*-IS*3000*, resembling the structures found in pZY-NDM1 and pNDM-Cf7308. This structure was a combination of Tn*3000* and Tn*125* remnants. Previous studies have described the prototype structures of Tn*3000* and Tn*125* associated with *bla*_NDM-1_, as vital vehicles for its dissemination, namely IS*3000*-*groEL*-*groES*-*cutA*-*dsbD*-*trpF*-*ble*_MBL_-*bla*_NDM-1_-IS*Aba125*-IS*3000* and IS*Aba125*-IS*CR27*-*groEL*-*∆groES*-*cutA*-*dsbD*-*trpF*-*ble*_MBL_-*bla*_NDM-1_-IS*Aba125*, respectively (Poirel et al., 2012; Campos et al., 2015). Compared with traditional Tn*3000* and Tn*125*, one copy of IS*3000* and IS*Aba125* was absent and another copy of IS*Aba125* was incomplete in pCFBC-1 (Figure 5A). The genetic background of *bla*_IMP-4_ was *intI1*-*bla*_IMP-4_-*Kl.pn.13*-*mobC*-IS*6100* (MGE1 of pCFBC2). Compared with the genetic context of *bla*_IMP-4_ in other plasmids (VR1 of Figure 5B), we observed its subtle changes in pCFBC-2, suggesting the occurrence of gene recombination. Class 1 integrons should be responsible for the transfer of *bla*_IMP_ gene. Thus far, *bla*_IMP-4_-associated class 1 integrons, including In809, 823, 823b, 1,377, 1,456, 1,460, and 1,589, has been reported in *Enterobacteriaceae* (Lee et al., 2017; Matsumura et al., 2017; Dolejska et al., 2018; Liang et al., 2018; Liu et al., 2021; Zhao et al., 2021). The distinction between In823 and In823b depends on the integrality of the *intI1* gene. Furthermore, we identified a large MGE2 situated between two fragments (*fipA∆1* and *fipA∆2*) in pCFBC2. In addition to containing the common Tn*6292* (IS2-IS26-*qnrS1*, IS*Kpn19* and *tnp* genes) (VR2 of Figure 5B), one *repB* gene, two toxin-antitoxin proteins (*higA* and *yafQ*), mobile elements (IS*5075*-∆Tn*3*-IS*26*) and a class 1 integron carrying gene cassettes *aac(6′)-Ib3* and *arr-3* were assembled on it. The interruption of the *fipA* gene has been reported could promote the accumulation of plasmids in diverse hosts and facilitate the aggregation of mobile elements (Yang et al., 2018), which was a beneficial explanation for the formation of this MGE2.

Phylogenetic analysis (Figure 6) was conducted to unveil evolutionary characteristics and homology of *C. freundii*. The results revealed that *C. freundii* BC73 clustered with *C. freundii* MEI002, *C. freundii* CAV1321, *C. freundii* 064C1, *C. freundii* CF8_ST22, *C. freundii* IDR1800045912-01-00, *C. freundii* P7699, *C. freundii* MH17-012 N and *C. freundii* DY2007. Interestingly, these isolates all belonged to the ST22 *C. freundii* strain and were distributed in different countries over the span of a decade, which suggested that the ST22 *C. freundii* strains have disseminated globally and they may be highly clonal. Notably, *C. freundii* BC73 co-carrying *bla*_NDM-1_ and *bla*_IMP-4_ and *C. freundii* DY2007 harboring *bla*_NDM-5_, isolated from dongyang, China in 2020 (Ye et al., 2023), were found to be the most closely related isolates. Previous report has described that the differences between *bla*_NDM-1_ and *bla*_NDM-5_ were represented by mutations at only two specific sites (88, 154) (Sun et al., 2019). Based on above findings, we proposed a bold hypothesis that *C. freundii* BC73 likely evolved from DY2007 through vertical propagation.

Since the initial discovery of NDM-1 and IMP-4 in *Enterobacteriaceae*, these carbapenemases have rapidly disseminated worldwide (Chu et al., 2001; Yong et al., 2009). In recent years, NDM-1 or IMP-4 producing *C. freundii* has frequently identified in the clinical setting, which further aggravated the concerns for public health (Wu et al., 2016; Liu et al., 2021). However, *C. freundii* with the coexistence of NDM-1 and IMP-4 has been rarely reported. To our knowledge, only one such isolate, named wang9, has been reported in China. The *C. freundii* wang9 isolate belonged to ST415, and the genes *bla*_NDM-1_ and *bla*_IMP-4_ were located on a conjugative IncHI1B plasmid, pwang9-1. The *bla*_NDM-1_ gene was located on the transposon Tn*AS3* (IS*91*-*sul*-IS*Aba14*-*aph (3′)-VI*-IS*30*-*bla*_NDM-1_-*ble*_MBL_-*trpF*-*dsbD*-IS*91*) while the *bla*_IMP-4_ gene was carried by integron In*1337* (*intI1*-*bla*_IMP-4_-*∆Kl.pn.13*-*qacG2*-*aac(6′)-Ib4*-*∆catB3*) (Qiao et al., 2023). However, the above characteristics were markedly distinct in our isolate. The more attention should be paid on further monitoring and genetic analysis of NDM-1 and IMP-4-producing *C. freundii* isolates and flexible transposition units for better understanding of multiple drug resistance transfer.

## Conclusion

In this study, we identified and characterized the genome of *C. freundii* BC73 co-carrying *bla*_NDM-1_, *bla*_IMP-4_ and *bla*_SHV-12_ from an inpatient with urinary tract infection after bladder cancer surgery. The *bla*_NDM-1_ and *bla*_IMP-4_ genes were located in a novel MDR plasmid and an IncN-IncU hybrid plasmid (pCFBC1, pCFBC2), respectively. In addition, multiple transposition units (ISs + resistant determinants), especially including two extensive transposition units (MGE1 in pCFBC1, MGE2 in pCFBC2), were found on it. The dissemination of NDM-1 and IMP-4-producing *C. freundii* isolates and ISs + resistant determinants should be of close concern in future clinical surveillance.

## Data availability statement

The datasets presented in this study can be found in online repositories. The names of the repository/repositories and accession number(s) can be found in the article/Supplementary material.

## Ethics statement

The ethical protocol was approved by the Ethics Committee of The Fifth Clinical Medical College of Henan University of Chinese Medicine (Zhengzhou People’s Hospital).

## Author contributions

NL: Data curation, Funding acquisition, Resources, Software, Visualization, Writing – original draft, Writing – review & editing. BT: Data curation, Funding acquisition, Software, Supervision, Writing – review & editing. HW: Software, Writing – review & editing. XiC: Software, Writing – review & editing. PW: Data curation, Resources, Writing – review & editing. ZW: Data curation, Writing – review & editing. XuC: Data curation, Writing – review & editing. XG: Supervision, Writing – review & editing. JG: Supervision, Writing – review & editing. YS: Funding acquisition, Supervision, Writing – review & editing.

## Footnotes

^1^ https://www.eucast.org/clinical_breakpoints/

^2^ http://pubmlst.org

^3^ https://cge.food.dtu.dk/services/ResFinder/

^4^ https://cge.food.dtu.dk/services/PlasmidFinder/

^5^ https://tool-mml.sjtu.edu.cn/oriTfinder/oriTfinder.html

^6^ https://bioinfo-mml.sjtu.edu.cn/ICEfinder/ICEfinder.html

^7^ https://tool2-mml.sjtu.edu.cn/VRprofile/VRprofile.php

^8^ http://crispr.i2bc.paris-saclay.fr/

^9^ https://blast.ncbi.nlm.nih.gov/Blast.cgi

^10^ https://www.ncbi.nlm.nih.gov
