# Supplementary material for: Coexistence of a novel NDM-1-encoding MDR plasmid and an IMP-4-encoding IncN-IncU hybrid plasmid in a clinical isolate of Citrobacter freundii BC73
Source: Front Microbiol. 2024 Jul 10;15:1388651. doi: 10.3389/fmicb.2024.1388651 (PMC11285197; doi:10.3389/fmicb.2024.1388651)
Supplement: Supplementary file 1 [file Data_Sheet_1.docx]

Supplementary Material

Coexistence of a novel NDM-1-encoding MDR plasmid and an IMP-4-encoding IncN-IncU hybrid plasmid in a clinical isolate of *Citrobacter freundii* BC73

Na Liu^1†^, Biao Tang^2†^, Hui Wang^1^, Xiangyang Chen^3^, Peipei Wen^1^, Zhaorui Wang^1^, Xu Chen^1^, Xiaobing Guo^4^, Jianjun Gou^4*^, Yinsen Song^1*^

^1^Translational Medicine Research Center, Zhengzhou People's Hospital, the Fifth Clinical College of Henan University of Chinese Medicine, Zhengzhou, China

^2^Key Laboratory of Systems Health Science of Zhejiang Province, School of Life Science, Hangzhou Institute for Advanced Study, University of Chinese Academy of Sciences, Hangzhou, China

^3^Department of Laboratory Medicine, Zhengzhou People's Hospital, the Fifth Clinical College of Henan University of Chinese Medicine, Zhengzhou, China

^4^Department of Laboratory Medicine, the First Affiliated Hospital of Zhengzhou University, Zhengzhou, China

†Contributed equally to the work

*** Correspondence:**Yinsen Song

E-mail: songys@hactcm.edu.cn

Jianjun Gou

E-mail: jianjung@zzu.edu.cn

**Supplement 1.**

The sequence of *bla*_NDM-1_ in pCFBC1 is as follows:

(64455-65267)

ctcagcg cagcttgtcg gccatgcggg ccgtatgagt gattgcggcg cggctatcgg gggcggaatg gctcatcacg atcatgctgg ccttggggaa cgccgcacca aacgcgcgcg ctgacgcggc gtagtgctca gtgtcggcat caccgagatt gccgagcgac ttggccttgc tgtccttgat caggcagcca ccaaaagcga tgtcggtgcc gtcgatccca acggtgatat tgtcactggt gtggccgggg ccggggtaaa ataccttgag cgggccaaag ttgggcgcgg ttgctggttc gacccagcca ttggcggcga aagtcaggct gtgttgcgcc gcaaccatcc cctcttgcgg ggcaagctgg ttcgacaacg cattggcata agtcgcaatc cccgccgcat gcagcgcgtc cataccgccc atcttgtcct gatgcgcgtg agtcaccacc gccagcgcga ccggcaggtt gatctcctgc ttgatccagt tgaggatctg ggcggtctgg tcatcggtcc aggcggtatc gaccaccagc acgcggccgc catccctgac gatcaaaccg ttggaagcga ctgccccgaa acccggcatg tcgagatagg aagtgtgctg ccagacattc ggtgcgagct ggcggaaaac cagatcgcca aaccgttggt cgccagtttc catttgctgg ccaatcgtcg ggcggatttc accgggcatg cacccgctca gcatcaatgc agcggctaat gcggtgctca gcttcgcgac cgggtgcata atattgggca attccatcaa

The sequence of *bla*_IMP-4_ in pCFBC2 is as follows:

(4061-4801)

atgagcaagt tatctgtatt ctttatattt ttgttttgta gcattgctac cgcagcagag cctttgccag atttaaaaat tgaaaaactt gatgaaggcg tttatgttca tacttcgttt gaagaagtta acgggtgggg cgttgttcct aaacatggtt tggttgttct tgtagatgct gaagcttatc taattgacac tccatttacg gctaaagata ctgaaaagtt agtcacttgg tttgtggaac gtggctataa aataaaaggc agtatttcct ctcattttca tagtgacagc acgggcggaa tagagtggct taattctcaa tccatcccca cgtatgcgtc tgaattaact aatgagctgc ttaaaaaaga cggtaaggtt caagctaaaa attcatttgg cggggttaac tattggctag ttaaaaataa aattgaagtt ttttatccag gcccaggaca cactccagat aacctagtag tttggctgcc tgaaaggaaa atattattcg gtggttgttt tattaaaccg tacggtctag gtaatttggg tgacgcaaat ttagaagctt ggccaaagtc cgctaaatta ttaatatcca aatatggtaa ggcaaaactg gttgttccaa gtcacagtga agctggagac gcatcactct tgaaacttac attagagcag gcggttaaag ggttaaacga aagtaaaaaa ccatcaaaac taagcaacta a
